# Risk factors and current state of therapy for anemia after kidney transplantation

**DOI:** 10.3389/fmed.2023.1170100

**Published:** 2024-01-08

**Authors:** Yan Tang, Jiayu Guo, Jiangqiao Zhou, Zijie Wan, Jinke Li, Tao Qiu

**Affiliations:** ^1^The Department of Organ Transplantation, Renmin Hospital of Wuhan University, Wuhan, Hubei, China; ^2^Department of Urology, Renmin Hospital of Wuhan University, Wuhan, Hubei, China

**Keywords:** kidney transplantation, anemia after transplantation, risk factors, intervention, research progress

## Abstract

Post-transplant anemia is one of the most common complications in kidney transplant recipients, severely affecting patient prognosis and quality of life, and is an independent predictor of graft kidney loss and patient mortality. However, our clinical understanding and the attention given to post-transplant anemia are currently insufficient. This paper reviews the current status, risk factors, and therapeutic progress in anemia after transplantation in kidney transplant recipients. We recommend that clinical staff pay attention to anemia and its complications in kidney transplant recipients and intervene early for anemia.

## 1 Introduction

Kidney transplantation is the most effective treatment for end-stage kidney disease (ESKD), and a successful kidney transplant can restore the patient’s kidney function to almost normal levels, including endocrine functions (1, 2). Post-transplantation anemia (PTA) is a common complication after kidney transplantation, and studies have shown that the incidence of PTA is 20–50% at different stages after transplantation (3–7). Although the vast majority of cases of PTA can be corrected in the early stages following successful kidney transplantation, there are still patients who progress to anemia or secondary anemia, which can seriously affect the recipients’ prognosis. PTA has been shown to reduce patient quality of life (8). Anemia has been linked with significant cardiovascular morbidity and mortality in renal transplant recipients (9, 10). Although anemia is a serious complication of transplantation, it has not attracted the attention of researchers. This paper therefore reviews the current status, risk factors, and available interventions for anemia after transplantation in kidney transplant recipients in order to prompt clinical workers to pay early attention to anemia after kidney transplantation.

## 2 Classification and diagnosis

Anemia describes a state in which the level of hemoglobin (Hb), the number of red blood cells, and/or the specific capacity of red blood cells within peripheral blood units is below the low normal limit (11). There is currently no exact staging or grading of the degree of anemia after transplantation (11). It is still generally graded using the normal human anemia scale now. In order to identify the effects of anemia on the post-transplant recipient and the transplanted kidney at different times, some centers have differentiated between PTA occurring within 6 months and PTA occurring after 6 months (12), as early and late anemia, respectively.

At present, according to the World Health Organization and the American Transplant Society, anemia is diagnosed in adults living at sea level with Hb ≤130 g/L for men, Hb ≤120 g/L for women, or Hb ≤110 g/L for pregnant women (13). According to the Kidney Disease: Improving Global Outcomes (KDIGO) initiative and the European Kidney Best Practice group, anemia is defined as Hb ≤120 g/L in men and menopausal women and Hb ≤110 g/L in non-menopausal women (14, 15). The reference range for hemoglobin concentrations in the blood may vary depending on the population analyzed, age, sex, environmental conditions, and dietary habits (16).

## 3 Prevalence and risks of anemia in kidney transplant recipients

### 3.1 Prevalence of anemia in kidney transplant recipients

Anemia is one of the most common complications in patients with CKD, with both the incidence and degree of anemia gradually increasing as renal function decreases. A study has found that more than 50% of cases of CKD are combined with anemia, while the prevalence of anemia in the uremia phase reaches 90.2% (17). A retrospective study of 649 samples taken in Mexico between 2013 and 2017 found an anemia prevalence of 73.1% in patients prior to kidney transplantation (18). Similarly, another retrospective study in Turkey found that before kidney transplantation, the prevalence of anemia and severe anemia reached 86.7 and 58.8%, respectively (19). Fortunately, kidney transplantation can improve the symptoms of anemia in patients with CKD to some extent. But due to intraoperative blood loss, repeated postoperative blood tests, infection, rejection, delayed recovery of transplanted kidneys, drugs and other factors (6, 20, 21), the incidence of anemia remains high in patients after kidney transplantation. Indeed, the incidence of PTA decreased significantly as kidney function improves after transplantation. The prevalence of PTA at 1, 3, 6, and 12 months after kidney transplantation has been reported as 84.3, 39.5, 26.2, and 21.6%, respectively (22). Since then, the incidence of PTA has remained at high level and even increased. In further studies, the prevalence of post-transplant anemia after kidney transplantation ranged from 25 to 41.4% (12, 23), with a 2-year PTA prevalence of 36.6%, while the incidence of anemia at 3, 5, and 10 years after transplantation was reported to be 41.5, 35.3, and 93.2%, respectively (24).

### 3.2 Risks of anemia in kidney transplant recipients

#### 3.2.1 Influence of anemia on cardiovascular function in kidney transplant recipients

Lower hemoglobin levels are associated with higher cardiovascular events (10). The annual incidence of cardiovascular disease (CVD) after kidney transplantation is 3.5–5%, which is 50-times that in the general population (25). Among the causes of death among kidney transplant recipients, CVD ranks first (40.9%) (26). Rates of cardiac death in renal transplant recipients (RTRs) are higher than in the general population, with the rate of cardiac death 10-times higher and the annual rate of fatal or non-fatal CV events 50-times that of the general population (27, 28). Anemia is an independent risk factor for clinical and echocardiographic cardiac disease, as well as mortality in end-stage renal disease patients (29). Kidney transplant recipients are considered to be a specific category of patients with CKD and are at risk for CKD-related complications (30). Long-term anemia causes hyperdynamic changes in the circulatory system and long-term overload of the heart and myocardial ischemia. This leads to anemic heart disease, as well as changes in heart rate, arrhythmias, changes in the structure of the heart, and congestive heart failure in severe cases (31). A retrospective cohort study of patients with no clinical heart disease who survived 1 year after kidney transplantation by a Canadian team showed that anemia was an important and major risk factor for left ventricular hypertrophy, 1 to 5 years after transplantation (10). However, most of these studies were observational or small intervention trials, which also focused on patients with CKD and did not take into account the poorer renal function and higher proteinuria in patients with PTA. Therefore, it is important to study whether positive treatment of anemia can improve CVD in PTA patients. However, studies have shown no benefit in correcting anemia or of high hemoglobin levels on cardiovascular disease or survival in CKD patients (32–35).

#### 3.2.2 Influence of anemia on kidney function in kidney transplant recipients

Previous studies have shown an association between anemia and adverse transplant outcomes, including graft failure, rejection and patient survival (36). During a specific physical examination of 18,383 healthy elderly people in Tokyo, Japan, it was found that patients with Hb <12 g/dL (male) or Hb <11 g/dL (female) had a 2.215- or 2.2-fold risk, respectively, of new CKD compared with individuals with normal Hb levels (37). In addition, these patients had 2.618-times the risk of deteriorating kidney function, respectively (37). A study in 385 kidney transplant recipients showed that both persistent anemia and late-onset anemia were associated with an increased risk of graft loss (36). Furthermore, studies such as that conducted by de Andrade have shown that patients with anemia have a 3.8-fold higher risk of losing a transplanted kidney than patients without anemia (38). It’s worth nothing that each increase in the degree of anemia increases the risk of graft loss by 2.77-times (hazard ratio [HR], 2.77; 95% confidence interval [CI], 1.50–5.13) (22). Studies such as that conducted by Jones have shown that patients with anemia have a 5.25-fold risk of transplant kidney failure compared with non-anemic patients (39). PTA also increases the incidence of post-transplant rejection, which is 1.8-times greater than in non-anemic patients (40). Previous studies have suggested that residual renal function is the most important predictor of PTA and that impaired renal function in renal transplant recipients is proportional to the severity of anemia (41). However, the role of correcting anemia on graft kidney function is not clear. A meta-analysis showed no difference between the ESA and no ESA groups (42). Similarly, in CKD patients, no nephroprotective effect of anemia correction has been observed (32). In contrast, the prospective study by Tsujita et al. showed that correcting anemia to target levels (12.5–13.5 g/dL) slowed the time to deterioration of renal function (43). These studies suggest that the occurrence of PTA after kidney transplantation is detrimental to the recipient’s transplant kidney function. We need to pay attention to the occurrence of PTA in the recipient at an early stage. The effectiveness and safety of anemia correction in improving graft outcomes in renal transplant patients remains to be further validated.

#### 3.2.3 Influence of anemia on survival and quality of life in kidney transplant recipients

Previous studies have demonstrated that anemia is associated with increased mortality and morbidity in patients with various diseases. For example, anemia is associated with shortened survival in patients with lung and cervical cancers (44), while severe anemia (Hb <11 g/dL) is consistently associated with high mortality (HR, 4.36; 95% CI, 3.04–6.27) (5). The development of anemia after kidney transplantation has a very significant impact on the recipient’s survival and is usually indirectly caused by the effect of anemia on other functions. However, due to the observational design of studies, causality cannot be affirmed. A retrospective study of 4,217 kidney transplant recipients in France found that all-cause mortality was as high as 6.8% in PTA recipients and 4.55% in the no-PTA group (23). The main reasons for this may be related to the type of the patient’s primary disease, the deterioration of the transplanted kidney function and the long-term use of immunosuppressive drugs. Anemia can also seriously affect the quality of life of kidney transplant recipients. For example, patients with anemia have been shown to experience chronic fatigue, decreased activity endurance, and cognitive decline, as well as increased length of hospital stay and costs associated with anemia (8, 45). Treatment of anemia may have some benefits on the quality of life of patients after kidney transplantation, but whether it will reduce the complications and mortality of patient needs further study.

## 4 Risk factors for the occurrence of PTA

### 4.1 Causes of anemia before kidney transplantation

Patients with ESKD often have varying degrees of anemia before surgery. The main reason for this is bone marrow suppression due to decreased erythropoietin (EPO) secreted by the renal interstitial cells and the accumulation of uremic toxins in the blood that inhibit the activity of EPO (46), resulting in decreased erythrocyte production. Additionally, the blood loss caused by hemodialysis or the abnormal iron metabolism caused by blood loss and microvascular inflammation can also lead to the occurrence of anemia (47). Patients with ESKD often use daily diet to control the production of toxins, and may also suffer from a loss of appetite because of the disease, while patients with end-stage kidney disease tend to have uremia toxins exceeding the level that causes catabolism, which may lead to maladaptation of nutrients and a lack of folic acid, vitamin B12, iron, and other hematopoietic substrate. This eventually triggers anemia (11). Anemia may occur in the context of bone metastases in kidney cancer, lupus nephropathy, and hemolytic uremic syndrome, as well as multiple myeloma and diabetic nephropathy (48–50). A retrospective analysis of 410 patients (Hb <10 g/dL) in South Korea found that those with 25-hydroxyvitamin D < 10 ng/dL before kidney transplantation had a higher risk of developing anemia than those with 25-hydroxyvitamin D ≥ 10 ng/dL, while vitamin D deficiency may also be a risk factor for anemia in patients with ESKD (21). A deficiency of levonidin has also been shown to be associated with anemia (51). The degree of anemia in pre-transplant patients affects the development of anemia in post-transplant patients to varying degrees (52). Therefore, the correction of anemia of patient needs to be considered before kidney transplantation and receive early post-transplant work-up.

### 4.2 Early causes of anemia after transplantation

Early anemia after kidney transplantation is often attributed to iron deficiency, blood loss, immunosuppression, and viral infections. Inadequate iron storage during transplantation, blood loss during surgery, increased iron utilization for compensatory production of red blood cells due to blood loss, and malnutrition can all contribute to iron deficiency (53). Moreover, frequent blood tests in the early postoperative period resulting in frequent small blood loss in patients can exacerbate the incidence and extent of anemia (54). Immunosuppressive drugs are usually used after kidney transplantation to prevent the occurrence of graft and receptor rejection; however, immunosuppression causes the patient’s entire immune system to be suppressed, inhibiting the bone marrow hematopoietic system and leading to the possibility of anemia. A study in Israel evaluating the incidence of anemia in pediatric patients with kidney transplantation and CKD showed that renal function recovered and glomerular filtration rates improved after kidney transplantation, but recipients had a higher incidence of anemia, possibly due to immunosuppressive therapy and EPO resistance (47). In a large cohort study enrolling 864 adult subjects, the prevalence of severe anemia immediately after kidney transplantation was 62.7%, significantly associated with anti-thymocyte globulin (ATG) /anti-lymphocytic globulin (ALG) administration (55). Simultaneously, the use of immunosuppressants decreases the body’s resistance to infection, resulting in various infections (56); cytomegalovirus (57, 58), and parvovirus B19 (59) typically occur early after transplantation, with a very low proportion and count of reticulocytes (60). Infection with parvovirus B19 must be strongly suspected when refractory and severe anemia with reticulocytopenia develops after transplantation (61). Studies have also shown that avoiding the use of steroids in the first 6 months after kidney transplantation also increases the incidence of PTA (62). Acute kidney injury and nutritional deficiencies are also important influencing factors for early PTA (5). Therefore, it is important to monitor the iron stores and immunosuppression status of patients in the early post-transplant period and make timely adjustments to avoid the development of PTA.

### 4.3 Cause for late PTA

The onset of late PTA is associated with impaired kidney graft function and the development of renal insufficiency (63). Delayed graft function, impaired kidney graft function, and acute rejection are risk factors for PTA (62). Serum creatinine and glomerular filtration rates have been shown to affect Hb in patients (20). For example, when eGFR >60 mL/min/1.73 m^2^, 18.7% of kidney transplant recipients experienced anemia, compared with only 2.4% of the general population. Further, when eGFR was 30–60 mL/min/1.73 m^2^, 34.8% of recipients developed anemia after kidney transplantation and 3.8% of the general population suffered from anemia (64). Therefore, renal transplant recipients are more susceptible to the influences of inadequate glomerular filtration rate, which leads to the development of PTA. We need to pay early attention to recipients with impaired kidney graft function and delayed graft function. In kidney transplant patients, kidney graft function deteriorates over time due to various factors. A multicenter study in France confirmed that deterioration of kidney graft function is a significant risk factor for PTA, and that renal transplant recipients have increased proteinuria early in the deterioration of kidney function (23). An increase in urine protein has been shown to be significantly associated with anemia (65). In addition, multiple studies have reported that a longer transplant time is an important influencing factor PTA (9, 23, 66).

### 4.4 Other causes of anemia after transplantation

Sex is a contributing factor in PTA, and previous studies have suggested a higher incidence of PTA in female kidney transplant recipients (19, 67), which may be associated with menstrual blood loss and resulting iron deficiency in women. Pre-menopausal women recipients may need more attention. In terms of age, pediatric transplant recipients may be more likely to develop PTA than adults (68), and in a multicenter study in Argentina, Hb levels were significantly correlated with age, as lower Hb levels in children (19, 69). At the same time, donor age has been suggested to be a risk factor for PTA (70). This may be because the donor kidney in older donors may have poorer endocrine function. At the same time, studies have pointed out that the non-use of EPO before transplantation is also a major predictor of PTA (66). There are other causes, such as a meta-analysis of 29,061 kidney transplant recipients that showed a significantly increased risk of anemia in patients receiving renin-angiotensin system (RAS) (71).

## 5 Status of interventions for anemia in kidney transplant recipients

Anemia is a syndrome, not a disease. Therefore, the cause must always be investigated, and treatment must be primarily for causal disorders (72). Based on published evidence, comprehensive anemia testing approximately 3 months after transplantation allows for prompt correction of anemia and treatment and improved prognosis (73). However, there are no specific recommendations for PTA treatment in kidney transplant recipients in KDIGO guidelines, and the current treatment of anemia after kidney transplantation is mainly based on CKD rational for the use of anemia-associated erythropoiesis stimulating agent (ESA), iron therapy, hypoxia-inducible factor prolyl hydroxylase inhibitors (HIF-PHI), and other (74, 75) (Table 1).

**Table 1:** Status of interventions for anemia in kidney transplant recipients.

Treatment	Reference	Interventions	Outcomes	Adverse effects
Erythrocyte-producing stimulating hormone	Pile et al. (76)	Epoetin beta: a starting dose of 50 U/kg once per week	Improvements in quality of life	NA
	Choukroun et al. (77)	Epoetin-β: start with a low dose weekly, dose escalation,	Less progress to ESKD; longer graft survival; lower cardiovascular events; improvements in quality of life	More return to dialysis and more death in low Hb-target group
	Heinze et al. (78)	Erythropoietin	High risk of mortality	Higher doses of erythropoietin with a higher incidence of cardiovascular, malignant, infection-related deaths
	Sánchez-Fructuoso et al. (79)	Erythropoietin receptor activator continuity	Better target Hb levels	Hypertension
	Budde et al. (80)	Epoetin α/βat: every 2–4 weeks for once; Continuous erythropoietin receptor activator: once-monthly	Stable Hb levels; good tolerability	Hemolytic anemia; pancytopenia; thrombocytopenia; angina pectoris; unstable angina; deep vein thrombosis; hypertension; injection site pain
	Bloom et al. (81)	Darbepoetin α: every 2 weeks for 24 weeks	Improved Hb levels; better HRQOL	Urinary tract infection; acute renal failure; nausea
Iron therapy	Iorember et al. (82)	Parenteral iron therapy: a single infusion of 1–2 mg/kg	Lower prevalence of early- and late-onset anemia; lower requirement for either ESA rescue or blood transfusion	NA
	Mudge et al. (83)	IV iron polymaltose: 500 mg single dose Oral ferrous sulfate: 210 mg elemental iron daily, continuously	Fewer gastrointestinal side-effects; fewer blood transfusions;	NA
	Rozen-Zvi et al. (84)	IV iron supplementation	Associated with increased Hb levels; lower rete of decline of eGFR	Chest pain; palpitations; weakness; nausea; dyspnea
Hypoxia-inducible prolyl hydroxylase inhibitor	Li et al. (54)	Roxadustat: orally three times a week	Improve Hb levels; good safety performance; stable renal function; no rejection	Symptoms of fatigue
Blood transfusion therapy	Ferrandiz et al. (85)	Blood transfusion	Higher incidence of DSAs	Rejection reaction
	Khedjat et al. (86)	Blood transfusion	No association with DSA, rejection graft loss; no influence with long-term outcomes	NA

HRQOL, health-related quality of life; IV, intravenous; DSAs, donor-specific antibodies; NA, not available; Hb, hemoglobin; ESKD, end-stage kidney disease.

### 5.1 Erythrocyte-producing stimulating hormone (ESA)

According to the National Kidney Foundation’s Kidney Disease Outcomes Quality Initiative, hemoglobin (Hb) levels below 11 g/dL are currently proposed treatment targets for anemia (87). KDIGO guidelines recommend initiating ESA therapy in patients with CKD only when Hb concentrations <10.0 g/dL and target Hb levels of 11.5 g/dL (14). Multiple evidence-based medical data show that ESA treatment significantly improves postoperative quality of life and reduces the need for blood transfusions (88). Recombinant human erythropoietin (rhEPO) is the first generation of ESA applied to the clinic, rhEPO is a short-acting preparation, 2 to 3 per week sub-administration; Daepotin α (DPO) belongs to the second generation of ESA. The mechanism of improving anemia is the same as that of rhEPO, but it is more biologically active and only needs to be administered once every 1 to 2 weeks; Persistent Erythropoietin Receptor Activator (CERA) is a third-generation ESA with advantages such as long half-life and low frequency of administration, but studies have confirmed its long-term efficacy and safety are inferior to other ESA (89). ESA is effective in correcting anemia and maintaining hemoglobin concentrations in the target range in most patients with CKD (90). Many post-transplant patients are anemic but only few receive adequate anemia treatment. Even transplant recipients with severe anemia received epoetin in only 17.8% of cases (12). Study showed that target hemoglobin level of 11.5 to 13.5 g/dL improves the vitality and mental health domains in quality of life Short Form (SF) - 36 scores and not associated with adverse changes in cardiovascular outcomes or increased cardiovascular morbidity or thrombotic events. However, treatment did not reduce the rate of decline in graft function (76). The main reason for this may be that some physicians believe that even without treatment, most patients will have symptoms of anemia improving on their own in the short term after surgery. Other studies have shown no effect of ESA on kidney function (91). It has also been reported that the treatment of EPO can cause complications such as hypertension and stroke, resulting in a poor prognosis for patients (92, 93). The laboratory evaluation of anemia including iron status and other substrates and replacement should be performed prior to treatment with ESA with a view to rational use of ESA for desired efficacy. The specific efficacy and influence of the use of ESA requires further study in the future.

### 5.2 Iron therapy

Iron is the basic ingredient for the synthesis of Hb, and studies have shown that early anemia after kidney transplantation is not due to the incomplete recovery of kidney function, but rather iron deficiency (94). Therefore, kidney transplant recipients with anemia after transplantation should routinely undergo measurement of serum ferritin, transferrin saturation, and other indicators, supplemented by serum hypersensitivity C-reactive protein and other deficit indicators if judged necessary. In addition, the causes of the iron deficiency should be investigated, and timely iron treatment administered according to the needs of patients, iron supplements before or after the kidney transplant for prevention might also be considered.

Iron therapy is typically divided into two categories: oral iron and intravenous iron. Commonly used forms of oral iron include ferrous sulfate, ferrous gluconate, and the newly emerged iron citrate, heme iron polypeptide, for example. The impact of oral iron on iron metabolism is close to the physiological state (95). However, the main disadvantages of oral iron are gastrointestinal adverse effects and the slow absorption of oral iron, which is not conducive to maximum iron utilization and rapid supplementation for patients in urgent need of iron supplementation. Intravenous iron agents include low-molecular weight dextran iron, iron sucrose, iron isomalt sucrose and iron carboxymaltose. The effectiveness and safety of intravenous iron in correcting renal anemia have been confirmed by a of evidence-based medical guidelines (96). However, the irregular application of intravenous iron can cause iron overload and damage to vital organs such as the liver and heart (97). Iron carboxymaltose, commonly used in intravenous iron preparations, was found to induce severe fibroblast growth factor 23-induced hypophosphatemia (98). Hence, we need to be vigilant about the amount of intravenous iron we use and avoid overloading. Gafter-Gvili et al. recommend that the use of an appropriate combination of erythropoietin stimulators and iron agents may be more beneficial in maintaining hemoglobin targeting at 12.5–13 g/dL in kidney transplant recipients (73). The Kidney Disease: Improving Global Outcomes (KDIGO) guidelines for the care of kidney transplant recipients recommend that anemia in kidney transplant patients should be monitored and treated in the same way as patients with CKD (99), while, CKD populations have indicated that an increased risk of stroke and venous thromboembolism when ESA therapy is used to target high Hb levels (32, 35, 100). CAPRIT (77) as the only clinical randomized cntrolled study evaluating the therapeutic target of PTA, divided Hb into normal group (130–150 g/L) and partially corrected group (105–115 g/L). The preservation of renal function and cardiovascular events in the normal group were better than those in the partially corrected group. Therefore, it is suggested that the therapeutic target of PTA is higher than that of other CKD patients.

### 5.3 Hypoxia-inducible prolyl hydroxylase inhibitor (HIF-PHI)

The safety of ESA in the treatment of anemia has been questioned after an increased risk of death, cardiovascular events, and stroke was observed in the ESA intervention trial (35). Therefore, compared with external supplementation of EPO, stimulating the production of endogenous EPO by other drugs might have higher safety and applicability. HIF-PHI is a novel oral small molecule drug class that stimulates endogenous EPO production and improves iron utilization (101). Four HIF-PHI agents, roxadustat, daprodustat, vadadustat and molidustat, have been investigated in clinical trials. Roxadustat was the first HIF-PHI to enter a Phase 3 clinical trial, and was recently approved for the oral treatment of anemia in China (102). Roxadustat has been shown to correct anemia and maintain hemoglobin levels in the presence of low ferritin saturation and a gradual decline in ferritin levels (103). Roxadustat has also been shown to significantly increase hemoglobin levels and has shown good safety, renal stability, and no rejection in patients with PTA (54). HIF-PHIs were not inferior to ESAs in correcting anemia when using the Hb increase from baseline to the evaluation period as the primary endpoint in most trials (104, 105). However, its high price limits its large application by most patients, while several side effects of roxadustat need to be noted, including gastrointestinal diseases, nasopharyngitis, and back pain (102). A recent meta-analysis showed a 31% higher risk of thrombosis events versus ESAs (105). At present, daprodustat and vadadustat are also approved for listing in Japan, but their effects and safety need to be verified by further clinical studies. In the absence of conclusive data on the reduction of cardiovascular risk with the use of HIF-PHI, their use to correct hemoglobin levels in transplant recipients should be treated with more caution. It is noted that no randomized controlled trial has been designed for kidney transplant recipients so far, given their metabolization by CYP enzymes, possible drug interactions in kidney transplant recipients should also be carefully evaluated.

### 5.4 Anti-infective and antiviral therapy

Kidney transplant recipients require long-term immunosuppressants after surgery, which primarily work by suppressing the body’s immune system, making patients more susceptible to bacterial and viral infection (106). Anemia due to chronic infection is often improved with anti-infective therapy. To prevent opportunistic infection in kidney transplant recipients, anti-infective and antiviral prophylaxis is recommended after kidney transplantation. In patients with microvirus B19 infection, intravenous immunoglobulin (IVIG) is required in addition to reduced exposure to immunosuppressive drugs, and high doses of IVIG do not aggravate anemia in patients (61, 107).

### 5.5 Adaptation of immunosuppressants

Many immunosuppressive drugs used after transplantation have potential myelosuppressive effects, and immunosuppressants such as tacrolimus and mycophenolate mofetil (MMF) have been reported to be a cause of PTA after kidney transplantation (108). Anemia has been reported to be more common in patients taking MMF and sirolimus (12). Some studies have shown no relationship between anemia and immunosuppression, which may be due to the already poorer function of the transplanted kidney in such patients (30). Current regimens for adjusting immunosuppressive therapy include switching to low-intensity immunosuppressants or reducing the dose of immunosuppressants (109). However, it is important to note that in the process of reducing immunosuppression intensity, the risk of rejection may be increased.

### 5.6 Blood transfusion

In non-emergency situations, blood transfusion therapy is generally not recommended for kidney transplant patients. Study has shown that donor-specific antibodies and antibody-mediated rejection (AMR) occurs in patients undergoing transfusion therapy after kidney transplantation (85). The probability of AMR is significantly higher than in non-transfusion patients. In addition, this might interfere with the patients’ opportunity to be re-transplanted. Therefore, a strategy of blood transfusion in the clinic should always be treated with caution. However, in cases where it is necessary, we still do not hesitate to carry out antibody clearance in conjunction with blood transfusions.

## 6 Discussion

PTA is a common complication after kidney transplantation and yet, despite its high prevalence, low treatment rate, and serious consequences, the condition has not currently attracted sufficient attention. Due to differences in the definition of anemia, ethnicity, follow-up time, and intervention factors, for example, the reported incidence of PTA varies greatly among research centers. Therefore, we need to explore and reach consensus on the assessment criteria applicable to anemia in renal transplant patients in the future.

The main risk factors associated with the development of PTA include transplanted kidney function, polypharmacy, and infection. Risk factors can either aggravate PTA or worsen the disease caused by PTA. There are no national or international detailed systematic reviews or guidelines for the treatment of PTA. There is also insufficient guidance for the diagnosis and treatment of PTA in kidney transplant recipients, while the target level of Hb after PTA treatment also remains controversial. Optimal treatment of PTA may be ambivalent, depending on the underlying cause; for example, infection caused by PTA requires reduced or even discontinued immunosuppressant therapy, while PTA caused by kidney rejection requires immunosuppression to be strengthened. The short-term effects of PTA on kidney transplant patients are unclear and reversible, but their long-term negative effects are known.

This paper reviews the current epidemiology status, risk factors and available interventions for PTA in patients with kidney transplantation. We hope that clinicians will pay attention to PTA after renal transplantation and that systematic guidelines for the prevention and management of PTA after renal transplantation will be available in the near future.

## Author contributions

YT and JG drafted the manuscript, undertook the systematic literature search. ZW and JL are conceived and designed the study. JZ and TQ obtained funding and supervised the study. TQ, JL, and JZ had important intellectual input. All authors have approved the final version of manuscript before submission.
